# Empirical Stochastic Model of Multi-GNSS Measurements

**DOI:** 10.3390/s21134566

**Published:** 2021-07-03

**Authors:** Dominik Prochniewicz, Kinga Wezka, Joanna Kozuchowska

**Affiliations:** Faculty of Geodesy and Cartography, Warsaw University of Technology, 00-661 Warsaw, Poland; kinga.wezka@pw.edu.pl (K.W.); joanna.kozuchowska.gik@pw.edu.pl (J.K.)

**Keywords:** GNSS, stochastic model, noise, multi-GNSS positioning, observation correlation

## Abstract

The stochastic model, together with the functional model, form the mathematical model of observation that enables the estimation of the unknown parameters. In Global Navigation Satellite Systems (GNSS), the stochastic model is an especially important element as it affects not only the accuracy of the positioning model solution, but also the reliability of the carrier-phase ambiguity resolution (AR). In this paper, we study in detail the stochastic modeling problem for Multi-GNSS positioning models, for which the standard approach used so far was to adopt stochastic parameters from the Global Positioning System (GPS). The aim of this work is to develop an individual, empirical stochastic model for each signal and each satellite block for GPS, GLONASS, Galileo and BeiDou systems. The realistic stochastic model is created in the form of a fully populated variance-covariance (VC) matrix that takes into account, in addition to the Carrier-to-Noise density Ratio (C/N${}_{0}$)-dependent variance function, also the cross- and time-correlations between the observations. The weekly measurements from a zero-length and very short baseline are utilized to derive stochastic parameters. The impact on the AR and solution accuracy is analyzed for different positioning scenarios using the modified Kalman Filter. Comparing the positioning results obtained for the created model with respect to the results for the standard elevation-dependent model allows to conclude that the individual empirical stochastic model increases the accuracy of positioning solution and the efficiency of AR. The optimal solution is achieved for four-system Multi-GNSS solution using fully populated empirical model individual for satellite blocks, which provides a 2% increase in the effectiveness of the AR (up to 100%), an increase in the number of solutions with errors below 5 mm by 37% and a reduction in the maximum error by 6 mm compared to the Multi-GNSS solution using the elevation-dependent model with neglected measurements correlations.

## 1. Introduction

The stochastic model of observations describes the dispersion of the measurement random errors (also referred to as measurement noise). Together with a functional model, expressing deterministic relationships between observations and unknown parameters, it forms the mathematical model of observation. The stochastic model is expressed by a variance-covariance (VC) matrix that defines the precision of observations through variance elements and their physical correlations through covariance elements. It is an essential element in the parameter estimation process for almost all measurement systems wherein the minimum variance estimator condition is assumed. Furthermore, the stochastic model is of particular importance in the case of Global Navigation Satellite Systems (GNSS) data adjustment, for which precise definition of a fully propagated VC matrix is difficult to obtain due to a multi-dimensional dependency of GNSS observations quality and the complex correlations existing between the measurements. In recent years, the joint processing of multi-system observations from Global Positioning System (GPS), GLONASS, Galileo and BeiDou (referred to as Multi-GNSS solution) has become the expected standard in precision positioning applications. The stochastic properties of the Multi-GNSS solution can vary significantly, which increase the importance of the correct definition of the VC matrix. Nevertheless, adopting homoscedastic or simplified models of variance and neglecting various correlations between the GNSS signals leads to a reduction in the positioning solution accuracy and reliability. These are crucial issues in relative positioning models using carrier-phase observations, in which correct integer carrier-phase ambiguity resolution (AR) is a necessary condition for precise positioning, since it defines the shape and size of the search space for the ambiguity estimation and contributes to the significance level and power of the validation tests. Unfortunately, nowadays the most common approach is the simplified definition of the stochastic model, which ignores the differences in accuracy and correlation between different GNSS systems.

The existing approaches to model the GNSS observation variance components (mostly adapted from the GPS system) use, among others, the following methods: satellite elevation-dependent variance exponential function [1,2] and cosecant variance function [3,4]; Signal-to-Noise Ratio (SNR)-dependent variance model [5,6,7], Carrier-to-Noise density Ratio (C/N${}_{0}$)-based model [8,9] with extensions: SIGMA-ϵ[10,11] and SIGMA-Δ [12] models; the ionospheric scintillation-dependent variance model [13]; the receiver tracking error-dependent model (e.g., variance function based on tracking jitters) [13,14]; multipath-dependent variance function [15]; User Ranging Accuracy (URA)-dependent variance function [16,17].

The variance models, as mentioned above, are mainly empirical models based on fitting a function that approximates the variance depending on the selected parameter. Among the methods for determining empirical stochastic models of GNSS observation noise, we can distinguish two main strategies: methods based on modeling observation residues from a zero-length baseline or a short baseline [18,19,20] and methods using combinations of both types of measurements [21,22]. An alternative to empirical determination of the VC matrix elements are iterative methods based on the Least-Square Adjustment residuals analysis. These methods use Variance Component Estimation (VCE) algorithms by means of: Minimum Norm Quadratic Unbiased Estimation (MINQUE) [23,24,25], Best Invariant Quadratic Unbiased Estimation (BIQUE) [26,27] and Least-Squares Variance Component Estimation (LS-VCE) [28,29]. The iterative methods require an initial VC structure to be assumed, often accomplished using variance functions as mentioned above (e.g., exponential function [30] or cosecant function [4]).

Among positioning models using multi-frequency observations or observations from multiple receivers, functional models are most often based on linear combinations of multi-epoch observations (e.g., single or double differences, ionosphere- or geometry-free combinations). In that case, in addition to mathematical correlations [31], the observations physical correlations are also apparent and should be taken into account to accurately capture the variances of observations and the covariances between them in a VC matrix. The physical correlations can be divided into: temporal correlations, mainly due to the atmosphere, multipath and the receiver itself [18,32,33,34,35]; spatial- (between channels) and cross-correlation between observation types (e.g., code and carrier-phase, between frequency) [36,37,38]. Determination of correlation parameters, depending on the type of correlation is based on following methods: exponential models [32,39], tropospheric turbulence theory-based models [40,41] and autoregressive moving average (AMRA) modeling of observation residuals [7,42]. Moreover, the VCE algorithms are also applied to study the covariance components of the GNSS observation VC matrix [43,44,45,46].

The models, additionally extended with the characteristics of residual errors (e.g., atmospheric residual errors), constitute a separate group of stochastic models. They are based on the assumption that residual observation errors not included in the functional model can be equivalently captured by the fully populated VC matrix [47]. These models, mainly dedicated to kinematic positioning, use a priori values of individual errors [48,49,50], the residual observations from previous epochs [51,52] or the atmospheric correction errors estimation determined from the network of reference stations [53].

Stochastic models for GPS and GLONASS measurements have been analyzed from the very beginning of their use [1,54,55,56,57,58]. The authors of these analyses, apart from developing the observation variance empirical models, pointed to the lack of cross-correlation between code and phase observations and the existence of significant correlations between the same type of observations at two frequencies (e.g., [56,59]). The conducted analyses also show significant time-correlations for the codes C1, P1 and P2 [18].

Apart from the numerous works related to the refinement of stochastic models for GPS and GLONASS observations, research on stochastic models of state-of-the-art Galileo and BeiDou systems has also been conducted in the recent years. These studies include analyses of single systems (e.g., variance of dual-frequency BeiDou B1/B2 code and phase observations [60], variance-covariance components as well as cross- and time-correlation parameters for dual-frequency [61] and triple-frequency BeiDou signals [4], a combination of BeiDou-2 and BeiDou-3 stochastic modeling [62], noise characteristic for five Galileo signals [63]), combined dual systems: GPS/Galileo [22], GPS/BeiDou [64], GPS/IRNSS [65] and triple systems GPS/Galileo/BeiDou [66,67,68] solutions as well as Multi-GNSS system solutions (e.g., [69,70]).

The analysis of the influence of various stochastic models on the results of the positioning model solution leads to the conclusion that their improvement (e.g., by changing the variance function or the diagonal VC matrix to the fully populated) affects: (a) the accuracy of the unknowns estimation—improvement in the accuracy of the horizontal baseline components at mm-level and height component up to a few centimeters [71,72]; (b) carrier-phase integer ambiguity resolution—improvement of AR success rate in GPS L1/L2 geometry-based relative positioning model up to 25% [30], improving the efficiency of the AR validation test up to 10% in the case of false alarm probability using ratio test [4,19]; (c) overestimation of the precision—neglecting the cross-correlation leads to a too optimistic estimate of the baseline solution precision [32,37,73].

All the above-mentioned scientific works and research results lead to the conclusion that the stochastic model is one of the crucial elements in the GNSS observations processing, and its correct definition has a significant impact on the quality of the obtained results. The previous analyses for multi-system solutions did not cover all aspects of stochastic modeling (determination of variance, cross- and time-correlation specific to a particular system/signal/satellite block) for each systems and its impact on the model solution (ambiguity resolution and positioning accuracy). The lack of uniform parameters for all GNSS systems results that one of the approaches taken in multi-system positioning is making the approximate assumptions for stochastic observation parameters or adapting parameters from the GPS system [74,75,76].

The aim of this work was to develop an individual, empirical stochastic model of all Mutli-GNSS code and carrier-phase observations in the form of a fully populated VC matrix that takes into account the physical correlations between measurements. The primary contributions of this paper are summarized as follows:

- The empirical stochastic model was determined individually for the GPS, GLONASS, Galileo and BeiDou systems for each signal and individual blocks of satellites using a 4th-degree Carrier-to-Noise density Ratio-dependent model.
- The developed empirical stochastic model was compared with the theoretical model describing the nominal precision of GNSS receiver performance due to thermal noise.
- The impact of the stochastic model elements on the positioning solution was examined based on the relative carrier-phase model for a very short baseline and Kalman Filter estimation method. The analysis considered the impact of including: different variance models, cross- and time-correlation characteristics and specific parameters for different satellite blocks.
- The comprehensive analysis of the positioning accuracy and ambiguity resolution results was performed for various GNSS systems and signals combinations using the developed empirical stochastic model and the standard elevation-dependent model.

The ultimate goal of the presented analyses was to improve the quality of the multi-system and multi-frequency solution by refining the empirical stochastic model of Multi-GNSS measurements.

The paper is organized as follows. In Section 2 we present GNSS measurement noise characteristic and its theoretical impact on observation quality. In Section 3 the empirical stochastic modeling methodology is presented, which allows to estimate variance and covariance components based on zero-length and very short baseline observations. In Section 4 the positioning model used to evaluate the developed empirical stochastic model is described. The applied estimation method using a modified Kalman Filter, considering the time-correlation of observations, is also outlined. In Section 5 the data set, results of stochastic modeling, the impacts on AR and positioning solutions for different scenarios are analyzed. Finally, in Section 6, the summary and concluding remarks are made.

The following symbols and notation for the analyzed systems and signals were used in the paper: for GNSS systems the following abbreviations were used: G for GPS, R for GLONASS, E for Galileo and C for BeiDou; observation codes for pseudorange and carrier-phase measurements were adopted according to RINEX 3.04 format [77]: GPS—C1C/L1C for L1 C/A, C1W/L1W for L1 P(Y), C2W/L2W for L2 P(Y), C2L/L2L for L2 CL, C5Q/L5Q for L5(Q); GLONASS—C1C/L1C for L1 C/A, C1P/L1P for L1 P, C2C/L2C for L2 C/A, C2P/L2P for L2 P, C3Q/L3Q for L3(Q); Galileo—C1C/L1C for E1 OS(C), C5Q/L5Q for E5a(Q), C7Q/L7Q for E5b(Q), C8Q/L8Q for E5ab AltBOC(Q), C6C/L6C for E6(C); BeiDou—C2I/L2I for B1-2(I), C7I/L7I for B2b(I), C6I/L6I for B3(I). The analyses performed for individual satellite blocks distinguished the following blocks and types: GPS Block IIR (type A with legacy antenna panel and type B with improved antenna panel [78]), IIR-M, IIF; GLONASS M and K1; Galileo IOV and FOC; BeiDou-2 GEO, IGSO and MEO.

## 2. GNSS Measurement Noise

GNSS measurements, like all other observables, are burdened with measurement errors. Most of the measurement errors, with the highest value, such as the orbital and clock errors or atmospheric delays, are included in the observation functional model and taken into account in a deterministic manner. The rest of the errors can be included in the observation model using the laws of probability as stochastic quantities [56]. Then, their random behavior is assumed, and their values and mutual correlations are described as variances and covariances in the observation VC matrix. A significant portion of these errors is highly dependent on the GNSS receiver itself, its components performance, and the finite precision of measurements. This section presents the theoretical relationship between the signal strength, the measurement noise and the nominal precision of GNSS receiver performance.

From the point of view of electrical signals processing, measurement random errors can be treated as thermal noise caused by random electrons movement in any of the electronic components of the GNSS receiver system with a temperature above 0 K [9]. Assuming thermal noise is white noise (with constant power spectral density) with a Gaussian distribution, its power for typical temperature T=290 K and GPS L1 signal bandwidth B=2 MHz can be expressed as [79]:

(1) $$P_{N}=kTB$$

where $k=1.3806\cdot 10^{-23}$ $W\cdot s\cdot K^{-1}$ is the Boltzmann constant. This results in a noise power of approx. $8\cdot 10^{-15}$ W (−141.0 dBW in decibels relative to one watt) or noise power spectral density of $N_{0}=-204.0$ dBW/Hz expressed per unit of bandwidth. However, the noise power value alone does not yield sufficient information about the signal quality and the resulting measurement errors. The key to the signal quality analysis is the received signal power, and its ratio to the noise power (referred to as SNR) when it is related to a given bandwidth ($P_{N}$) or C/N${}_{0}$ if referred to the power per unit of bandwidth ($N_{0}$).

Table 1 summarizes a simplified example of received signal power budget and thermal noise for C/A GPS observation with the minimum transmitted signal power of about 26.8 W. The received signal power on the earth’s surface can be calculated after taking into account: the power path loss due to the spreading of the total signal energy over the entire surface area, satellite antenna gain caused by the amplification of the signal power towards the Earth in relation to the isotropic antenna, atmospheric attenuation and receiver antenna gain taking into account effective area of an omnidirectional receive antenna and gain of patch antenna relative to the isotropic. The presented values refer to the worst-case scenario, for a satellite elevation of about $e=5^{\circ }$, for which the values of satellite signal spread angle of $27.8^{\circ }$ and gain of the receiver antenna related to the isotropic one of −2 dBic were adopted [7,79,80].

**Figure 1:**
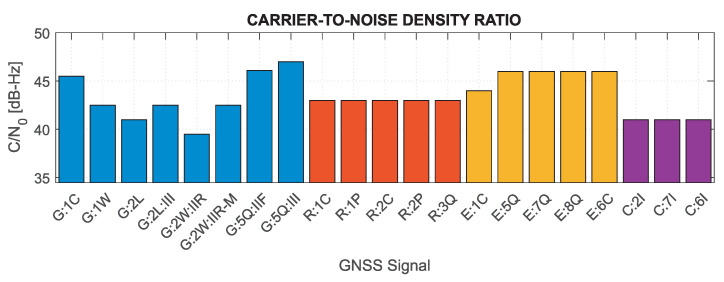
Nominal C/N${}_{0}$ for minimum received signal power for GNSS signals.

The numbers in Table 1 for a very low elevation satellite illustrate the lower values of the received signal power that can be acquired. This signal power relates directly to the minimum received signal power that is a guaranteed minimum received power level, as specified in the Interface Control Document (ICD) for each of the GNSS systems, but may vary slightly depending on the signal power budget assumptions. Based on the minimum received signal power, compiled in [81], the nominal (theoretical) C/N${}_{0}$ for GNSS signals were determined. Figure 1 shows the C/N${}_{0}$ for individual systems and signals analyzed in the study, distinguishing between individual satellite blocks for GPS signals. Note that if the information on the power of a given signal was not publicly available (as in the case of GLONASS P-code or BeiDou 6I), values for other signals of the given system were adopted for the calculation. Based on the presented values, it can be concluded that the nominal signal quality differs significantly between systems, reaching the lowest values for GPS 2L, 2W and BeiDou signals, which may considerably affect the GNSS receiver performance.

The SNR parameter describes the relative magnitude of the measurement noise and is therefore directly related to the precision of the code and carrier-phase observations. The nominal precision of GNSS receiver performance due to thermal noise can be calculated as tracking error in Delay Lock Loop (DLL) for code and in Phase Lock Loop (PLL) for carrier-phase measurements, given by [79]:

(2) $$\begin{array}{cc} \sigma_{C}^{2} & =\frac{B_{\mathrm{DLL}}d}{2{C/N}_{0}}\left(1+\frac{1}{t_{\mathrm{DLL}}{C/N}_{0}}\right)\;{[\mathrm{PRN}\mathrm{chip}}^{2}] \end{array}$$

(3) $$\begin{array}{cc} \sigma_{L}^{2} & =\frac{B_{\mathrm{PLL}}}{{C/N}_{0}}\left(1+\frac{1}{2t_{\mathrm{PLL}}{C/N}_{0}}\right)\;{[\mathrm{rad}}^{2}] \end{array}$$

where: $\sigma_{C}^{2}$ and $\sigma_{L}^{2}$ are tracking error variance for code and carrier-phase respectively, $B_{\mathrm{DLL}}$ and $B_{\mathrm{PLL}}$ are code and phase tracking loop bandwidth in Hz, $t_{\mathrm{DLL}}$ and $t_{\mathrm{PLL}}$ are DLL and PLL predetection integration intervals in seconds and *d* is the dimensionless correlator spacing normalized with respect to one Pseudorandom Noise (PRN) code chip. The C/N${}_{0}$ in these equations must be expressed in units of ratio-Hz.

**Figure 2:**
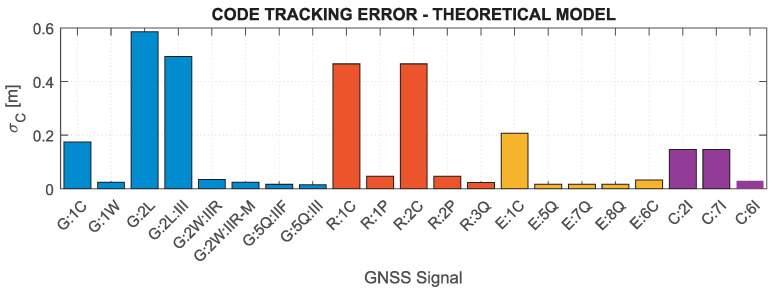
Nominal code tracking errors for GNSS signals for receiver Septentrio PolaRx5.

**Figure 3:**
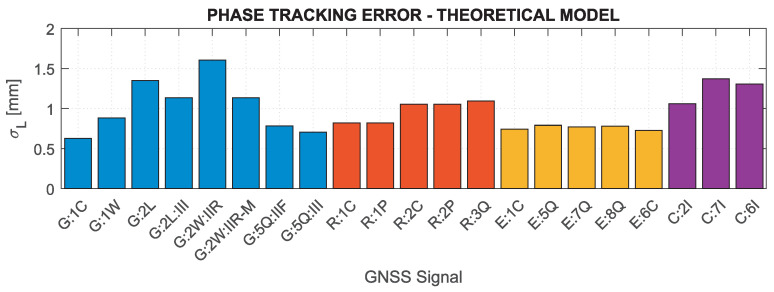
Nominal carrier-phase tracking errors for GNSS signals for receiver Septentrio PolaRx5.

Based on the above formulas, assuming the C/N${}_{0}$ determined for the minimum received signal power and the characteristics of the tracking loops for the Septentrio PolaRx5 receiver, which was used in the measurement experiment ($B_{\mathrm{DLL}}=0.25$ Hz, $B_{\mathrm{PLL}}=15$ Hz, $t_{\mathrm{DLL}}=0.10$ s, $t_{\mathrm{PLL}}=0.01$ s, d=0.1), the nominal errors of GNSS measurements were determined. Figure 2 and Figure 3 show theoretical tracking errors for code and phase observations, respectively, for signals corresponding to those shown in Figure 1. The carrier phase observation errors accurately reflect the variability of C/N${}_{0}$—the lower the value, the greater the error. For code observations, high error values of GPS 2L and GLONASS 1C and 2C signals are distinguished. This is mainly caused by the narrow nominal bandwidth of these signals, which is generally smaller than the actual transmission bandwidth [81].

Based on the results in Figure 1, Figure 2 and Figure 3, it can be concluded that the nominal measurement noise of individual observations varies significantly, and, assuming the same observation accuracy for different systems and signals, may cause significant inaccuracies of the realistic noise characteristics in the stochastic model.

## 3. Empirical Stochastic Modeling Methodology

In Section 2 we showed that the nominal measurement noise could be significantly different for different types of GNSS signals. To determine the actual value of the measurement noise and to create a realistic stochastic model of observations, the empirical stochastic modeling method based on the observation residuals was used. The empirical models of observation variance, as well as the time- and cross-correlation parameters, were derived using double-differenced (DD) and triple-differenced (TD) measurements from short baseline (SB) and zero-length baseline (ZB) conducted by two receivers of the same type. The time series of the mentioned measurement combinations allow to precisely separate the influence of particular correlations from each other. Thus, the SB TD combination was used to model the dependence of the carrier-phase and code variances on the C/N${}_{0}$ parameter. The time- and cross-correlation were determined based on the ZB DD observation time series analysis, as this combination is not burdened with the influence of multipath, does not de-correlate the measurements and allows to determine both parameters directly. This section presents the methodology of determination of individual parameters of the empirical stochastic model, the accuracy analysis of the created variances functions and the values of correlation coefficients based on daily observations for all systems and signals.

The simplified code (*C*) and carrier-phase (*L*) observation equations in units of length for a receiver *a* and a satellite *i* generalized for frequency *f* are given by:

(4) $$\begin{array}{cc} C_{a}^{i} & =R_{a}^{i}+\delta T_{a}^{i}+\delta I_{a}^{i}+K_{a,C}-K_{C}^{i}+\delta M_{a,C}^{i}+c\left(\delta t_{a}-\delta t^{i}\right)+\epsilon_{a,C}^{i} \end{array}$$

(5) $$\begin{array}{cc} L_{a}^{i} & =R_{a}^{i}+\delta T_{a}^{i}-\delta I_{a}^{i}+K_{a,L}-K_{L}^{i}+\delta M_{a,L}^{i}+c\left(\delta t_{a}-\delta t^{i}\right)+\lambda N_{a}^{i}+\epsilon_{a,L}^{i} \end{array}$$

where: *R* is the geometric satellite-receiver range, which includes satellite orbit error, satellite and receiver antenna phase center errors and carrier-phase wind-up bias, δT and δI are troposphere and ionosphere delays, $K_{C}$ and $K_{L}$ are hardware biases for a receiver (with a subscript *a*) and a satellite (with a superscript *i*) for code and phase measurements respectively, $\delta M_{C}$ and $\delta M_{L}$ are code and phase multipath, δt is a satellite or receiver clock error, *N* denotes integer carrier-phase ambiguity and $\epsilon_{C}$ and $\epsilon_{L}$ are code and carrier-phase random errors. All quantities, except for clock errors given in seconds and ambiguity given in carrier wave cycles, are expressed in units of length. Additionally, *c* is the speed of light, and λ denotes the carrier wavelength. Assuming the same atmospheric delays affect observations from two receivers for SB and ZB setup, the DD code and carrier-phase residuals can be calculated by subtracting from the observations the double differences of geometric distance and carrier-phase ambiguity, which can be easily determined, if the coordinates of both receivers are known with high accuracy. The TD residuals, derived as the time difference of two DD for consecutive observation epochs ($t_{1}$ and $t_{2}$), also significantly reduce the multipath error as it has almost the same value for two close epochs (e.g., for 1 Hz data).

Table 2 shows the calculation formula, the expectation (E{·}) and dispersion (D{·}) values for two linear residuals combination (DD and TD) of carrier-phase observations for the short and zero-length baseline, where $\tilde{L}\left(t_{1}\right)$ is the phase residual at epoch $t_{1}$ and symbol ∇Δ denotes the double-differencing operator. This example illustrates only phase observation residuals, but the formulas are the same for the code residuals (except for the carrier-phase ambiguity which is not existent in code observations). Formulas are presented in a generalized form (omitting the indexes of satellites and receivers) for intra-system differences (no differences are computed between two GNSS systems). It is assumed that two receivers of the same type are used to create differences, thus the measurement noise affecting observations from both receivers have the same magnitude. This prerequisition allows to eliminate the inter-frequency bias occurring in the differences computed using observation on a different frequency (as in GLONASS system, which uses Frequency Division Multiple Access (FDMA)).

The dispersion of observation residuals of linear combinations represents the measurement noise with variance $\sigma_{L}^{2}$ for phase observation and takes into account two types of correlations: time-correlation $\rho_{t}$ which affects the TD dispersion when time differencing is performed and cross-correlation $\rho_{\Delta }$ between observations from two receivers [21,22]. For the SB setup $\rho_{\Delta }$ can be neglected; however, for the ZB, when two receivers use the same antenna, the large part of the noise is the same due to the common antenna Low-Noise Amplifier (LNA) [45,82], which results in a significant cross-correlation of measurement from the receivers. When the observations from both receivers are burdened with common LNA noise, it is eliminated in the observation difference, significantly reducing the dispersion.

**Figure 4:**
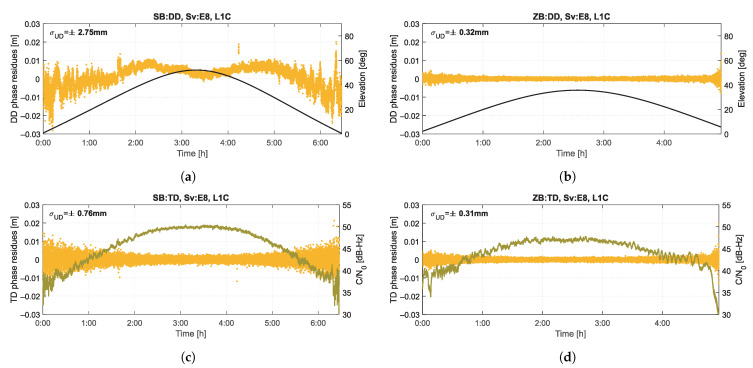
Time series of carrier-phase residuals of Galileo L1C signal from Sv8 for combinations: SB DD (**a**), ZB DD (**b**), SB TD (**c**), ZB TD (**d**).

Figure 4 presents the time series of phase residuals for four observation combinations listed in Table 2, for exemplary one of the processed signal—Galileo L1C from the Sv8 satellite. The satellite with the highest elevation for a given epoch was used as a reference satellite, so it can be assumed that its noise was constant and its impact on the presented results was minimized. The SB length was 4.75 m. Based on these series, one can see an apparent multipath effect in the DD combination for SB, which is eliminated for the TD combination and ZB observations. There is also a clear dependence on the satellite elevation (marked on top graphs for DD) and the C/N${}_{0}$ parameter (marked on bottom graphs for TD). The magnitude of the measurement noise clearly decreases for observations from the ZB, which is confirmed by the standard deviation of the time series, calculated for a uniform undifferenced (UD) level, amounting to ±0.76 mm, ±0.32 mm and ±0.31 mm, respectively for SB TD, ZB DD and ZB TD combinations. This confirms the existence of significant correlations that must be taken into account to correctly determine the measurement noise variance based on the presented combinations of observations.

In order to determine the variance of carrier-phase and code observations, the SB TD combination was used. The created empirical model of variance is based on fitting a function that approximates the variance depending on the C/N${}_{0}$ parameter. The advantage of choosing the C/N${}_{0}$ parameter, unlike the elevation angle, is a close theoretical relationship with the measurement noise (expressed by Equations (2) and (3)) and the possibility of including the actual received signal power. The relationship with the elevation is not direct [80]. Most of the modern precise GNSS receivers output C/N${}_{0}$. It is then available to users via RINEX files or RTCM and NMEA data streams. Unfortunately, for some types of receivers the parameter is unavailable.

Based on a general form of tracking error, Equations (2) and (3) can be represented as:

(6) $$\sigma =a_{1}\cdot 10^{-{C/N}_{0}/20}+a_{2}\cdot 10^{-{C/N}_{0}/10}$$

where: coefficients $a_{1}$ and $a_{2}$ are related to tracking loop bandwidths, predetection integration intervals and correlator spacing separately for code and phase measurements; C/N${}_{0}$ is expressed in dB-Hz. The developed empirical variance model uses a modified Equation (6) which extends to the form of a n=4 degree series, in the form:

(7) $$\sigma =\sum_{i=1}^{n}a_{i}\cdot 10^{\left(1-i\right){C/N}_{0}/40}$$

Function (7) is fitted to the standard deviation of the SB TD code and phase observations residuals determined for the adopted time series segments, and the coefficients $a_{i}$ are determined by the Least-Squares Method. To assess the variance of the observation noise ($\sigma_{L}^{2}$ and $\sigma_{C}^{2}$), the time-correlation of observation residues should be taken into account (according to Table 2)—the method of determining $\rho_{t}$ is described in the further part of this subsection.

**Figure 5:**
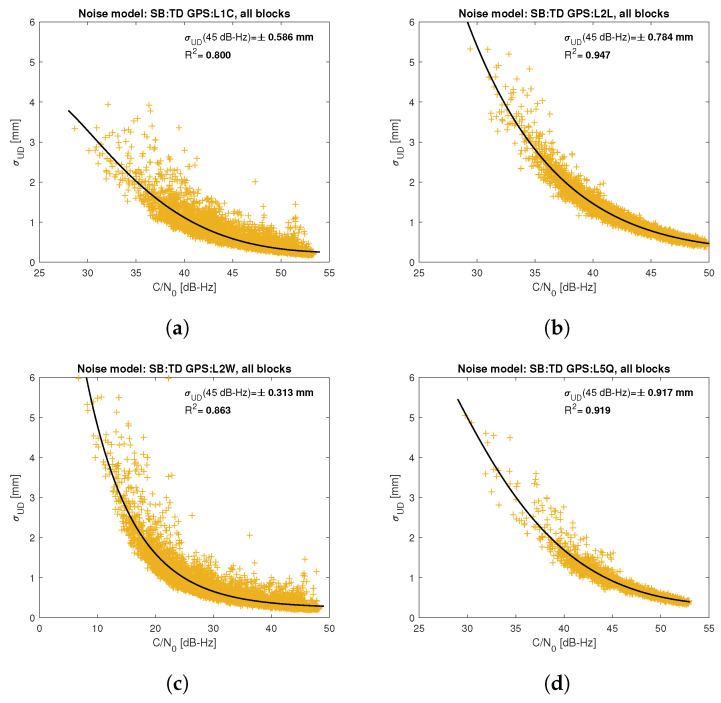
Empirical variance model for SB TD GPS carrier-phase measurements for signals: L1C (**a**), L2L (**b**), L2W (**c**), L5Q (**d**).

**Figure 6:**
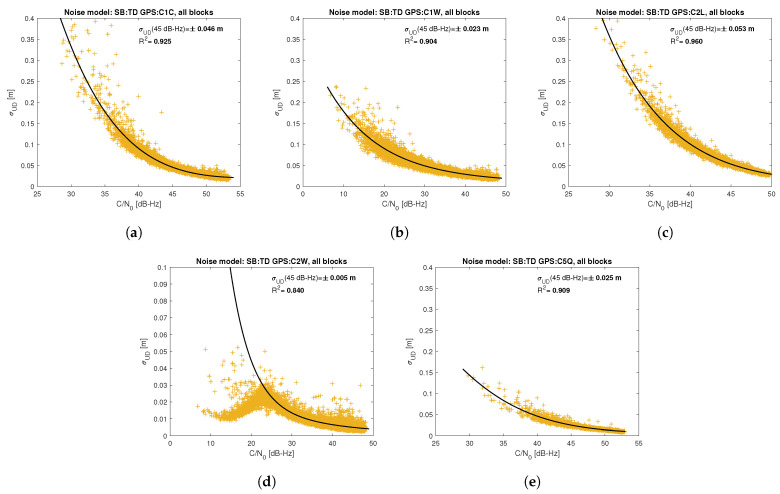
Empirical variance model for SB TD GPS code measurements for signals: C1C (**a**), C1W (**b**), C2L (**c**), C2W (**d**), C5Q (**e**).

**Figure 7:**
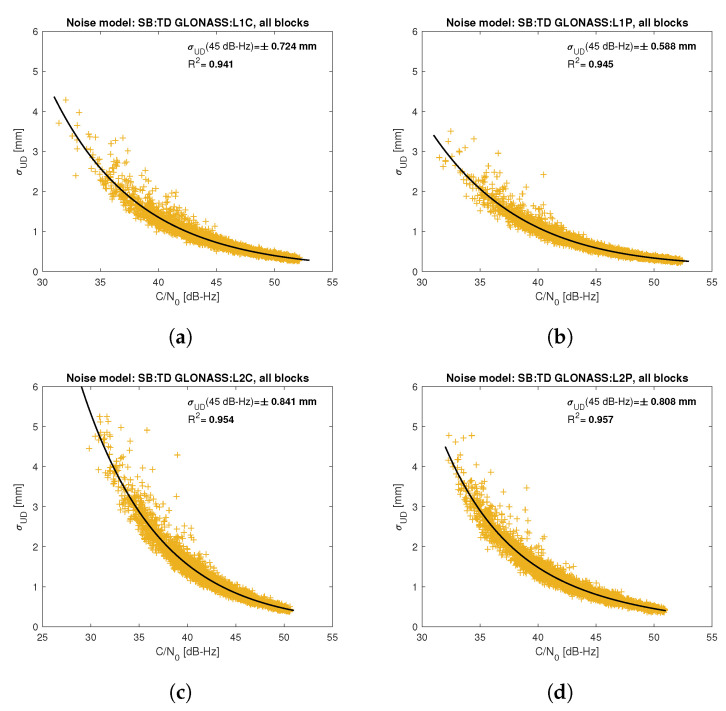
Empirical variance model for SB TD GLONASS carrier-phase measurements for signals: L1C (**a**), L1P (**b**), L2C (**c**), L2P (**d**).

**Figure 8:**
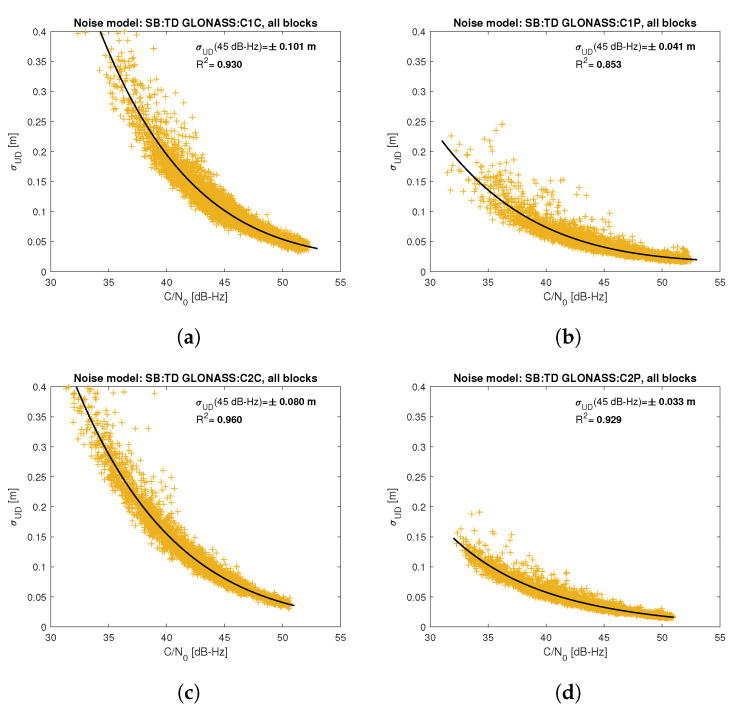
Empirical variance model for SB TD GLONASS code measurements for signals: C1C (**a**), C1P (**b**), C2C (**c**), C2P (**d**).

**Figure 9:**
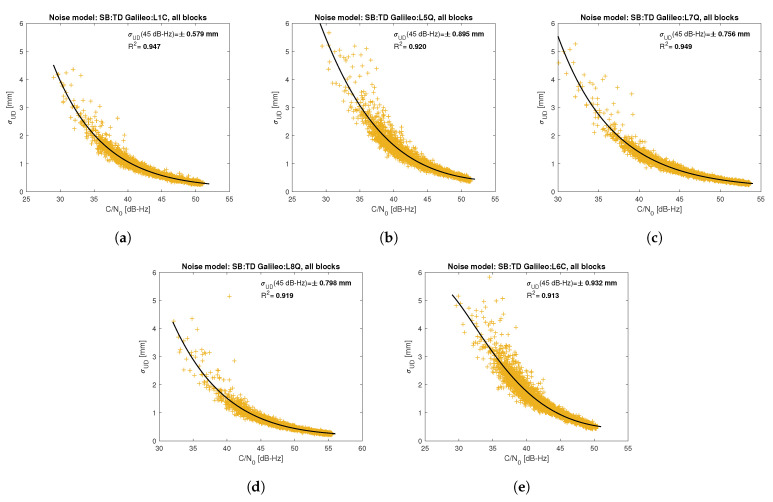
Empirical variance model for SB TD Galileo carrier-phase measurements for signals: L1C (**a**), L5Q (**b**), L7Q (**c**), L8Q (**d**), L6C (**e**).

**Figure 10:**
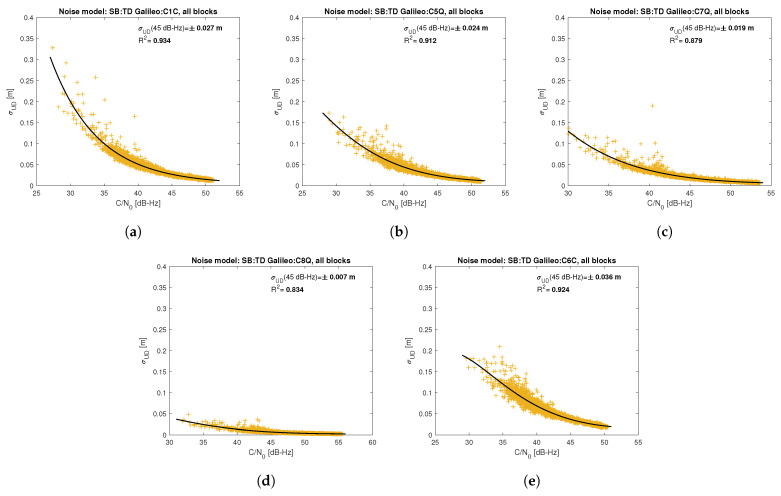
Empirical variance model for SB TD Galileo code measurements for signals: C1C (**a**), C5Q (**b**), C7Q (**c**), C8Q (**d**), C6C (**e**).

**Figure 11:**
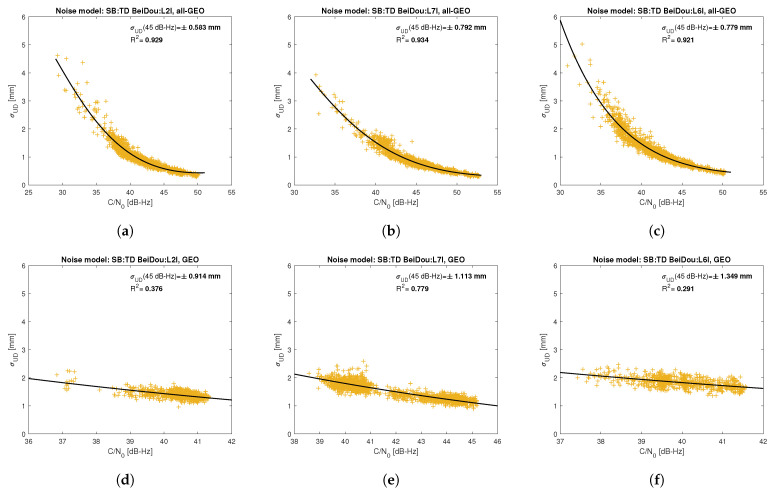
Empirical variance model for SB TD BeiDou carrier-phase measurements for signals: L2I (**a**), L7I (**b**), L6I (**c**) for all satellite blocks except for GEO satellite; L2I (**d**), L7I (**e**), L6I (**f**) for GEO satellites.

**Figure 12:**
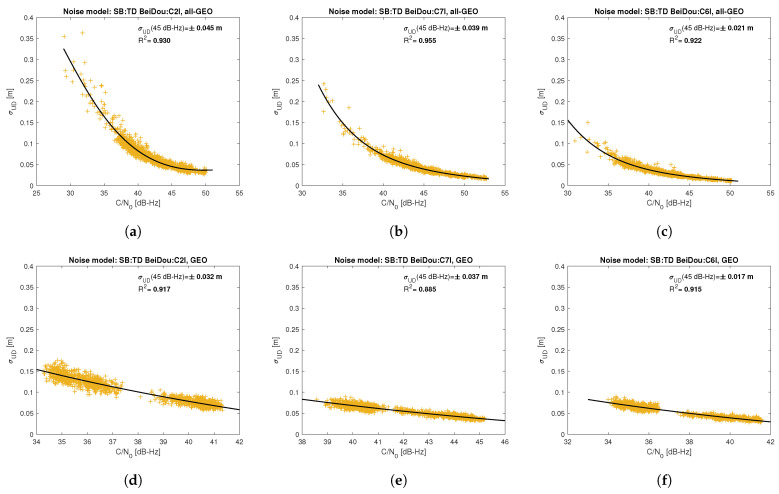
Empirical variance model for SB TD BeiDou code measurements for signals: C2I (**a**), C7I (**b**), C6I (**c**) for all satellite blocks except for GEO satellite; C2I (**d**), C7I (**e**), C6I (**f**) for GEO satellites.

Figure 5, Figure 6, Figure 7, Figure 8, Figure 9, Figure 10, Figure 11 and Figure 12 show the results of the approximation of the standard deviation of SB TD code and phase observations for all analyzed GNSS signals using the function (7). Standard deviations were determined for daily observations with an interval of 1 Hz for 120-s observation segments and recalculated to a UD level. The argument of the approximating function C/N${}_{0}$ was calculated as a mean value of carrier-to-noise density ratios of the reference satellite and the second satellite in a pair. A detailed description of the data used can be found in Section 5. Aforementioned Figures present the modeling results for all observations for a given signal with no distinction between satellite blocks. The exception is the BeiDou system, for which the n=2 degree model was adopted for observations from geostationary (GEO) satellites.

Based on the presented figures, it can be concluded that the selected approximating function accurately reflects the dependence of measurement noise standard deviation on the C/N${}_{0}$ parameter. The Figures also show the coefficient of determination $R^{2}$, which is within the range of 0.80–0.96, proving that the model replicates the empirical standard deviation values very well. The only exception is the model for GPS C2W code observations, for which the standard deviation of the noise has a non-standard pattern—it increases along with the value of C/N${}_{0}$ to about 25 dB-Hz and then decreases. This effect may be related to a signal power loss while tracking the P(Y)-code using the Z-tracking technique [83]. Nevertheless, this hypothesis cannot be confirmed unequivocally. The matter should be investigated in more detail. In this case, we use the same approximating function as for the other signal modeling only for observation with C/N${}_{0}>25$ dB-Hz. It should be noted, however, that the observation error is much smaller than for other code observations (in all the graphs, the same scale was adopted for the code noise variance with a maximum of 0.4 m, except for C2W observations for which the maximum is 0.1 m), and the impact on the final results of the positioning model solution is significantly smaller than the one of the carrier-phase measurements. The second exception is the model for BeiDou GEO observations. The low variability of the C/N${}_{0}$ for geostationary satellites makes precise modeling difficult—for GEO L2I and L6I signals, the differences of C/N${}_{0}$ for the determined carrier-phase variances are only 4 dB-Hz.

The time- and cross-correlation were determined based on the ZB DD observation time series analysis. To determine the time correlation, the autocorrelation function (ACF) for time series was used that measures the correlation between y(t) and y(t+τ) of y(t) stochastic process given by [18]:

(8) $$\sigma^{2}\left(\tau \right)=\frac{1}{T}\sum_{t=1}^{T-\tau }\left[y\left(t\right)-\bar{y}\right]\left[y\left(t+\tau \right)-\bar{y}\right]$$

where $\sigma^{2}\left(\tau \right)$ is the sample covariance of time series y(t) with itself at time lag τ, $\bar{y}$ denotes the mean of the time series *y* and *T* is the effective sample size of *y*. The time-correlation coefficient for lag τ is computed as:

(9) $$\rho_{t}\left(\tau \right)=\frac{\sigma^{2}\left(\tau \right)}{\sigma_{0}^{2}}$$

where $\sigma_{0}^{2}$ is a sample variance of the time series for τ=0. For the ZB DD combination, the variance of the time series is burdened with the influence of correlation $\rho_{\Delta }$, but assuming that the LNA noise is constant over time, it will burden the $\sigma^{2}\left(\tau \right)$ and $\sigma_{0}^{2}$ equally, so as a result, it does not affect the $\rho_{t}\left(\tau \right)$.

**Figure 13:**
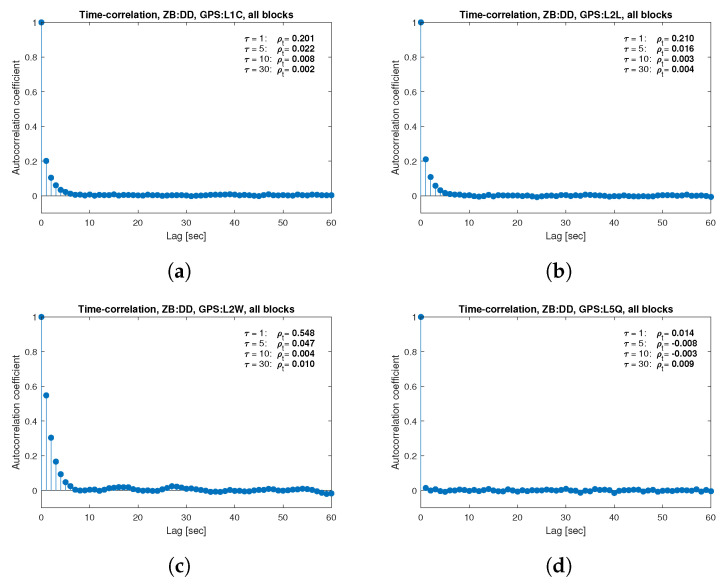
Sample autocorrelation coefficient for ZB DD GPS carrier-phase measurements for signals: L1C (**a**), L2L (**b**), L2W (**c**), L5Q (**d**).

**Figure 14:**
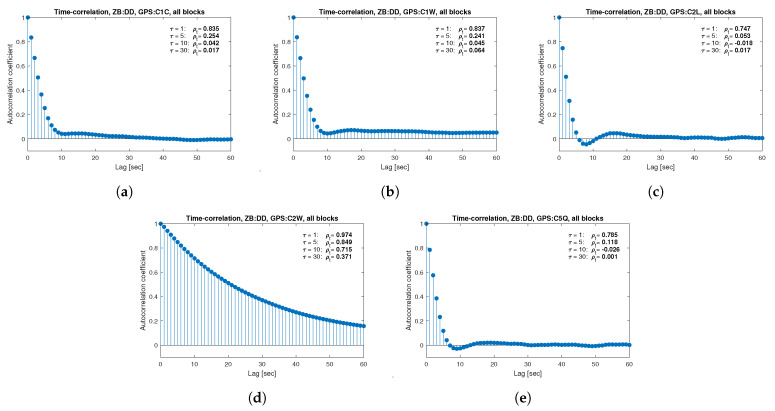
Sample autocorrelation coefficient for ZB DD GPS code measurements for signals: C1C (**a**), C1W (**b**), C2L (**c**), C2W (**d**), C5Q (**e**).

**Figure 15:**
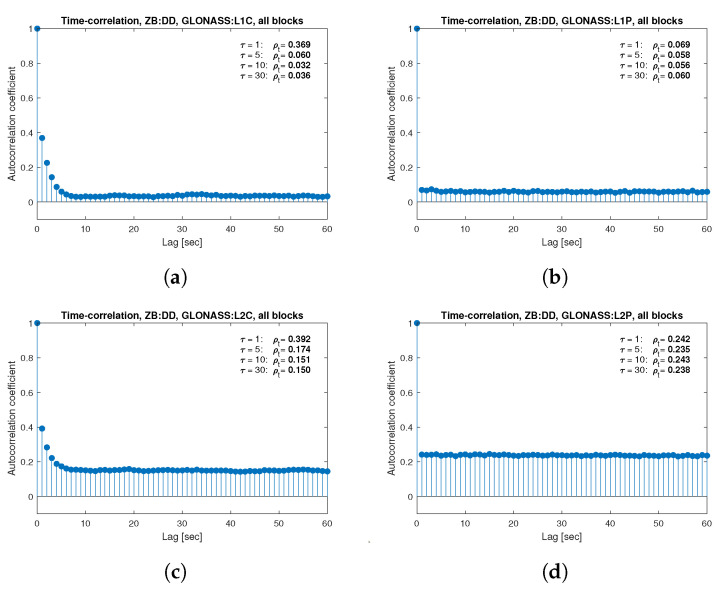
Sample autocorrelation coefficient for ZB DD GLONASS carrier-phase measurements for signals: L1C (**a**), L1P (**b**), L2C (**c**), L2P (**d**).

**Figure 16:**
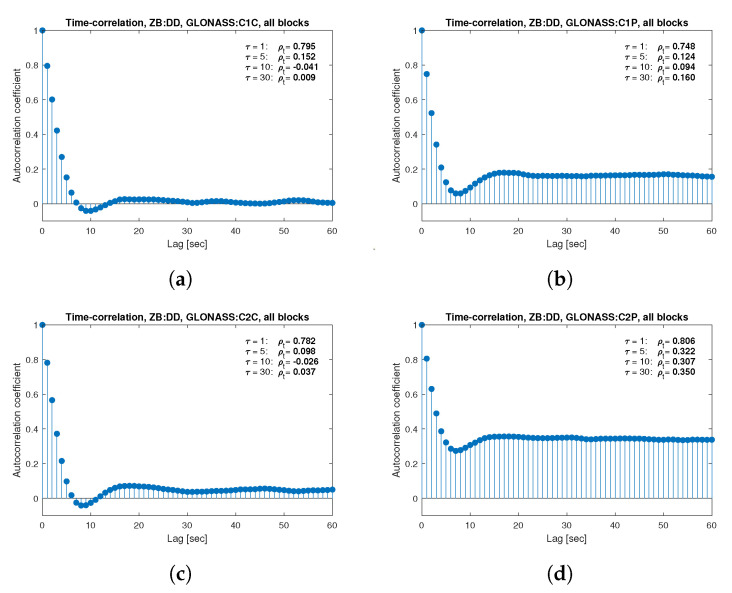
Sample autocorrelation coefficient for ZB DD GLONASS code measurements for signals: C1C (**a**), C1P (**b**), C2C (**c**), C2P (**d**).

**Figure 17:**
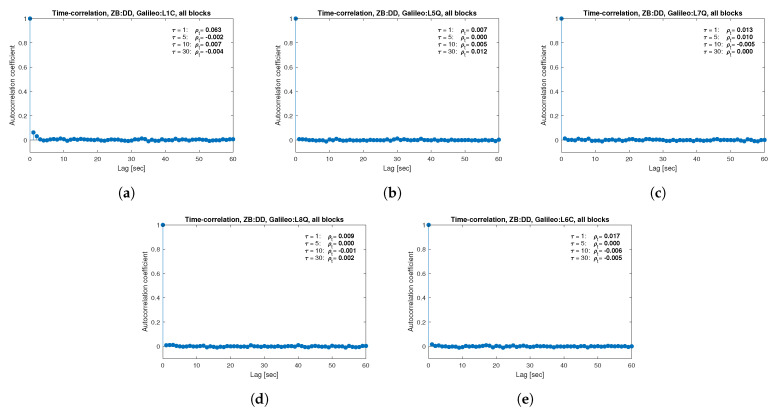
Sample autocorrelation coefficient for ZB DD Galileo carrier-phase measurements for signals: L1C (**a**), L5Q (**b**), L7Q (**c**), L8Q (**d**), L6C (**e**).

**Figure 18:**
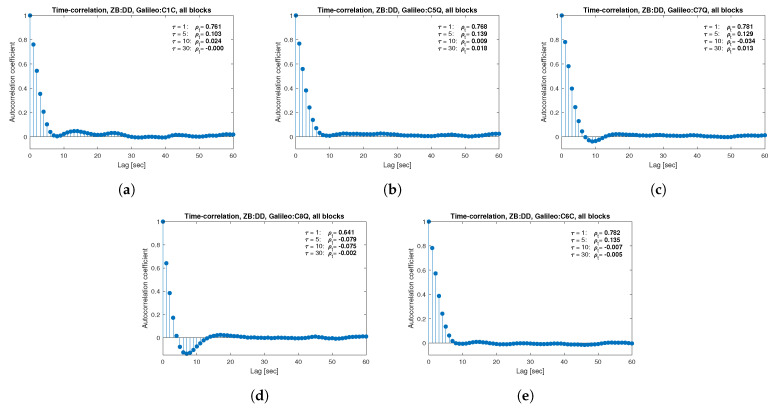
Sample autocorrelation coefficient for ZB DD Galileo code measurements for signals: C1C (**a**), C5Q (**b**), C7Q (**c**), C8Q (**d**), C6C (**e**).

**Figure 19:**
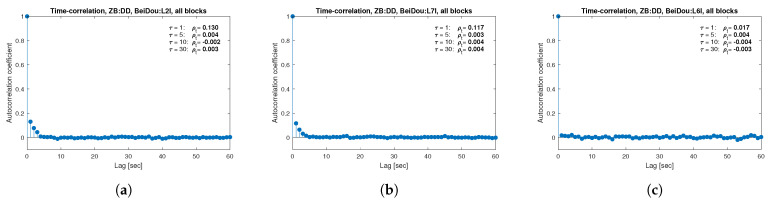
Sample autocorrelation coefficient for ZB DD BeiDou carrier-phase measurements for signals: L2I (**a**), L7I (**b**), L6I (**c**).

**Figure 20:**
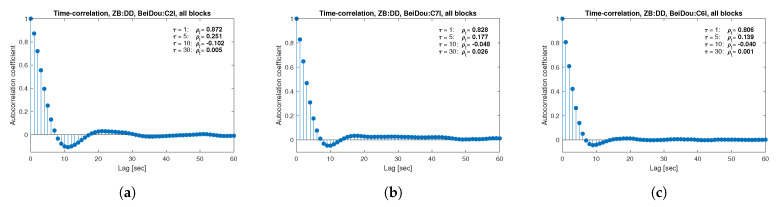
Sample autocorrelation coefficient for ZB DD BeiDou code measurements for signals: C2I (**a**), C7I (**b**), C6I (**c**).

Figure 13, Figure 14, Figure 15, Figure 16, Figure 17, Figure 18, Figure 19 and Figure 20 show the sample autocorrelation coefficient for code and phase observations for all analyzed GNSS signals based on daily 1 Hz measurements. As for the variance model analysis presented previously, the presented ACFs did not distinguish between satellite blocks and were calculated as averages for all satellites. The average $\rho_{t}$ for the selected lags (1, 5, 10 and 30 s) were also listed in the graphs. As can be easily seen, the time-correlation of the code observations reaches significant values for τ<10 s for all signals. There is also a noticeable high temporal correlation of pseudo-observation of encrypted GPS L2W and GLONASS C1P and C2P codes, for which this coefficient has significant values for the entire range of analyzed lags (τ≤60 s). For carrier-phase measurements, the time-correlation coefficient is much smaller, especially for new systems Galileo and BeiDou as well as for the GPS L5Q signal, for which for τ=1 s it immediately drops off to about zero. For the remaining carrier-phase measurements, the time-correlation is significant only for τ≤3 s except for GLONASS L2C and L2P observations where the $\rho_{t}$ remains constant regardless of the lag. These examples lead to the conclusion that for the analyzed signals, there is a significant time-correlation of observations, especially for 1 Hz measurements, and its neglect may cause essential simplification in the realistic definition of the Multi-GNSS observation stochastic model.

The cross-correlation coefficients of measurements were determined based on the covariance of two time series $y_{1}$ and $y_{2}$ according to the formula:

(10) $${\mathrm{cov}}_{y_{1},y_{2}}=\frac{1}{T-1}\sum_{t=1}^{T}\left[y_{1}\left(t\right)-{\bar{y}}_{1}\right]\left[y_{2}\left(t\right)-{\bar{y}}_{2}\right]$$

The correlation coefficient is then calculated as:

(11) $$\rho_{y_{1},y_{2}}=\frac{{\mathrm{cov}}_{y_{1},y_{2}}}{\sigma_{y_{1}}\sigma_{y_{2}}}$$

where $\sigma_{y_{1}}$ and $\sigma_{y_{2}}$ are standard deviation of $y_{1}$ and $y_{2}$, respectively.

**Figure 21:**
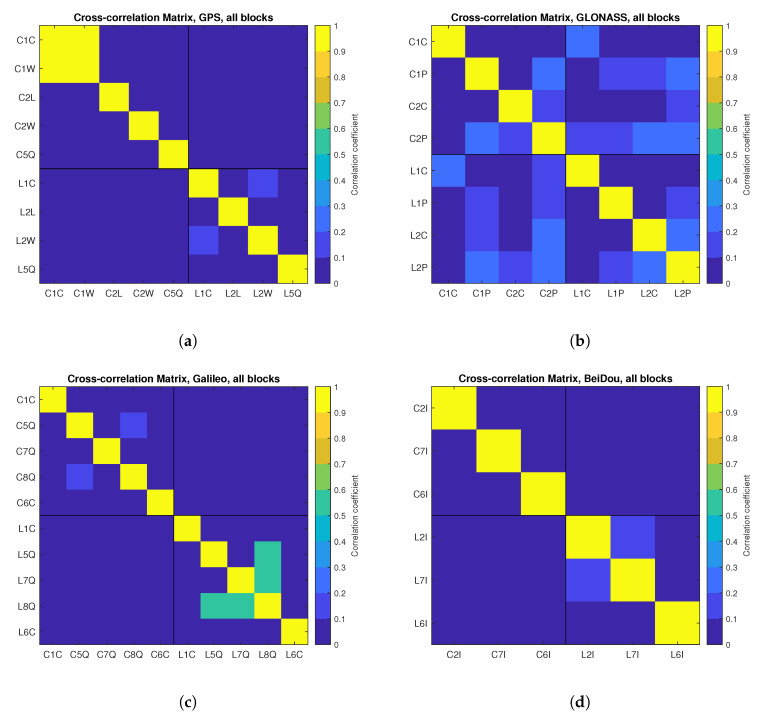
Cross-correlation coefficient matrix for ZB DD measurements for systems: GPS (**a**), GLONASS (**b**), Galileo (**c**), BeiDou (**d**).

The cross-correlation coefficients were determined between corresponding ZB DD time series of the observations to the same satellites for different types of signals and frequencies within one GNSS system, and the average for all satellites was taken as the final value. Figure 21 shows the cross-correlation coefficients of observations presented in a form of a correlation matrix for individual observation types for 1 Hz daily data. This Figure shows that only for the GPS C1C/C1W and Galileo L5Q/L7Q/L8Q measurements, we observe a significant cross-correlation between the observations. In the first case, it is due to a use of the Z-tracking technique of encrypted P(Y) code in which the difference measurements C1C-C1W are acquired instead of the direct observations of C1W [83,84]; this leads to a cross-correlation of observations reaching 0.95. In the second case, high correlation (up to 0.55) is a result of the structure of the Galileo E5 signal, which can be tracked independently for the E5a and E5b components or as a single wide bandwidth E5ab signal. In other cases, slight correlations between GPS L1C/L2W, GLONASS C1P/C2P and L2C/L2P, Galileo C5Q/C8Q measurements reaching 0.15 can be observed. No correlation was found between code and carrier-phase observations to the same satellites for all systems, except for the GLONASS system. However, in that case, when correlation coefficients are lower than 0.25, it can be concluded that the correlation is very weak and insignificant.

Summing up, it can be stated that the used combinations of observation residuals and the adopted fitting function allow for precise modeling of the dependence of the code and carrier-phase observation variance on the C/N${}_{0}$ parameter. The determined time- and cross-correlation coefficients may reach non-negligible values and significantly differ depending on the analyzed signal.

## 4. Positioning Model

Quantitative evaluation of the empirical, realistic stochastic model of GNSS observation was made based on the comparison of positioning model solution results that adopted different stochastic parameters. The geometry-based double-differenced code and carrier-phase observations positioning model for multi-frequency and Multi-GNSS systems was used for the tests with the a priori known atmospheric delays (i.e., the ionosphere-fixed troposphere-fixed model). The linearized functional and stochastic formulas of positioning models are given by:

(12) $$\begin{array}{cc} E\{z\} & =Hx \end{array}$$

(13) $$\begin{array}{cc} D\{z\} & =V \end{array}$$

where z is the observed minus computed DD carrier-phase and the code observation vector, H is the design matrix for position and ambiguities and x is a vector of unknown real-valued receiver position and integer-valued DD ambiguities. The stochastic model is captured by the dispersion with VC matrix V of DD observations. As the functional model used is widely described in the literature (e.g., [53,85,86]) it is not defined in detail here. It should only be noted that only the intra-system differences were generated for a multi-system solution without combining observations from different GNSS systems.

The fully populated VC matrix of the undifferenced measurements for one receiver *a* at a single epoch, denoted as $V_{0,a}$, is composed from matrices describing the variance C and cross-correlation Γ of observations in the form:

(14) $$V_{0,a}=\left[\begin{array}{cccc} C_{f_{1},a}^{2} & \Gamma_{f_{1},f_{2}}C_{f_{1},a}C_{f_{2},a} & \cdots & \Gamma_{f_{1},f_{m}}C_{f_{1},a}C_{f_{m},a}\\ \Gamma_{f_{1},f_{2}}C_{f_{1},a}C_{f_{2},a} & C_{f_{2},a}^{2} & \cdots & \Gamma_{f_{2},f_{m}}C_{f_{2},a}C_{f_{m},a}\\ \vdots & \vdots & \ddots & \vdots \\ \Gamma_{f_{1},f_{m}}C_{f_{1},a}C_{f_{m},a} & \Gamma_{f_{2},f_{m}}C_{f_{2},a}C_{f_{m},a} & \cdots & C_{f_{m},a}^{2} \end{array}\right]$$

where f=1,2,⋯,m denotes the type of signal/frequency/system. The variance and cross-correlation matrices for i=1,2,⋯,k satellites are computed using empirical stochastic models of variance (Equation (7)), time-correlation (Equation (9)) and cross-correlation (Equation (11)) parameters:

(15) $$\begin{array}{cc} C_{f,a} & ={\left(1-\rho_{t,C_{f}}\right)}^{-\frac{1}{2}}\cdot \mathrm{diag}\left(\sigma_{a,C_{f}}^{i=1},\sigma_{a,C_{f}}^{i=2},\cdots ,\sigma_{a,C_{f}}^{i=k}\right) \end{array}$$

(16) $$\begin{array}{cc} \Gamma_{f_{1},f_{2}} & =\rho_{f_{1},f_{2}}I_{k} \end{array}$$

where $I_{k}$ denotes identity matrix of size *k*. In this simplified notation, it was assumed that the same satellites were observed for all signals, but in a general case for a different number of satellites, the appropriate dimensions of the matrices should be considered. The time-correlation coefficient $\rho_{t}$ in the Equation (15) refers to the correlation between the epochs making up the TD combinations used in the variance model. In the VC matrix for a single epoch, the time-correlation between the observations used in the positioning model cannot be taken into account; this parameter was included in the Kalman Filter for the multi-epoch approach.

The final formulation of VC matrix for the DD model for *a* and *b* receivers is given by:

(17) $$V=D\left(V_{0,a}+V_{0,b}\right)D^{T}$$

where D is a block diagonal of *m* single-differencing matrices [87].

The solution of the positioning model presented by Equations (12) and (13) was employed by the Kalman Filter (KF). For the discrete-time standard KF, the prediction step at epoch *t* is performed as [88]:

(18) $$\begin{array}{cc} {\hat{x}}_{t}\left(-\right) & =F_{t-1}{\hat{x}}_{t-1}\left(+\right) \end{array}$$

(19) $$\begin{array}{cc} P_{t}\left(-\right) & =F_{t-1}P_{t-1}\left(+\right)F_{t-1}^{T}+Q_{t-1} \end{array}$$

where ${\hat{x}}_{t}$ is the estimate of $x_{t}$, $F_{t-1}$ is the state transition matrix from epoch t−1 to *t*, P is the state error covariance matrix and Q is the covariance matrix of a process noise; symbols (−) and (+) denote values before and after the measurement updating, respectively. For a continuous solution where the position and ambiguity are determined as unknowns, the state transition matrix is the identity matrix; the initial parameters for the process noise covariance matrix are described in the next section.

To handle the time-correlated errors in a multi-epoch solution the modified KF based on the time-differencing alghorithm (proposed by [89] and revised by [90]) is used. In this approach, the temporal correlation data, correlated with previous measurements at a time lag τ, is limited in updating step by differencing redundant information determined using correlation coefficient:

(20) $$z_{t}^{*}=z_{t}-\Psi_{t}z_{t-1}$$

where $\Psi =\mathrm{diag}(\rho_{t}\left(\tau \right))$ is the transition matrix of the time-correlated errors and contains correlation coefficient for time lag τ (see Equation (9)) [91]; superscript ${}^{*}$ denotes a modified time-differenced quantity. Hence the modified design matrix, modified measurement VC matrix R as well as the new correlation matrix describing correlation between measurement noise and process noise S are given by formulas:

(21) $$\begin{array}{cc} H_{t}^{*} & =H_{t}-\Psi_{t}H_{t-1}F_{t-1}^{-1} \end{array}$$

(22) $$\begin{array}{cc} S_{t} & =Q_{t-1}{\left(F_{t-1}^{-1}\right)}^{T}H_{t-1}^{T}\Psi_{t}^{T} \end{array}$$

(23) $$\begin{array}{cc} R_{t}^{*} & =R_{t}+\Psi_{t}H_{t-1}F_{t-1}^{-1}Q_{t-1}{\left(F_{t-1}^{-1}\right)}^{T}H_{t-1}^{T}\Psi_{t}^{T} \end{array}$$

The measurement VC matrix must be presented as a function of white (uncorrelated) measurement noise VC matrix N and time correlated noise VC matrix M in the form:

(24) $$R_{t}=M_{t-1}+N_{t}+\Psi_{t}N_{t-1}\Psi_{t}^{T}$$

and the decomposition of V into M and N can be done as [88]:

(25) $$\begin{array}{cc} M_{t} & =\Psi_{t}V_{t}\Psi_{t} \end{array}$$

(26) $$\begin{array}{cc} N_{t} & =V_{t}-M_{t} \end{array}$$

The solution for the updated state is then computed using a gain matrix K defined as:

(27) $$\begin{array}{cc} K_{t} & =\left[P_{t}\left(-\right){\left(H_{t}^{*}\right)}^{T}+S_{t}\right]\times {\left[H_{t}^{*}P_{t}\left(-\right){\left(H_{t}^{*}\right)}^{T}+R_{t}^{*}+H_{t}^{*}S_{t}+S_{t}{\left(H_{t}^{*}\right)}^{T}\right]}^{-1} \end{array}$$

(28) $$\begin{array}{cc} {\hat{x}}_{t}\left(+\right) & ={\hat{x}}_{t}\left(-\right)+K_{t}\left[z_{t}^{*}-H_{t}^{*}{\hat{x}}_{t}\left(-\right)\right] \end{array}$$

(29) $$\begin{array}{cc} P_{t}\left(+\right) & =P_{t}\left(-\right)-K_{t}\left[H_{t}^{*}P_{t}\left(-\right){\left(H_{t}^{*}\right)}^{T}+R_{t}^{*}+H_{t}^{*}S_{t}+S_{t}^{T}{\left(H_{t}^{*}\right)}^{T}\right]K_{t}^{T} \end{array}$$

The modified KF was used to obtain the float solution (approximate coordinates and real-valued DD ambiguities) with its VC matrix. The final baseline solution was obtained after fixed integer ambiguity using Integer Least-Squares (ILS) estimates and updating float solution. Note that the presented algorithm taking into account the time-correlation of observations can be simply reduced to a standard Kalman Filter when a time-correlation coefficient is assumed to be zero. A detailed description of the data set used to evaluate the empirical stochastic model impact on the positioning model solution is presented in Section 5.

## 5. Experiment Design and Test Results

To determine and test the empirical stochastic model of GNSS observations, measurements from two reference stations WUT1 and WUT2 located on the roof of Warsaw University of Technology Main Building (approx. location: $52^{{}^{\circ}}13^{\prime }15\prime \prime$ N, $21^{{}^{\circ}}00^{\prime }37\prime \prime$ E) were used. Seven subsequent daily session data sets (year 2017, DOY: 337-343) from WUT1 were used to derive the ZB combination, while another subsequent seven daily sessions (year 2018, DOY: 168-174) from both stations were used to calculate the SB combination for 4.75 m baseline. The same set of two Septentrio PolaRx5 receivers was used for both tests, and the measurements were carried out with a 1 Hz interval for the $0^{\circ }$ elevation mask. The precise Center for Orbit Determination in Europe (CODE) Multi-GNSS-EXperiment (MGEX) orbits were used for all calculations [92]. Note that the interval between the two periods did not affect the obtained results, as the observed constellation did not change during this time. However, the presented analyses do not include the BeiDou-3 constellation, which started operating in 2018. All calculations and analysis related to stochastic modeling and positioning solution were performed using in-house MATLAB software.

### 5.1. Empirical Stochastic Model

According to the methodology presented in the Section 3, based on the observations from the zero-length and short baseline, an empirical model of observation variance, as well as the coefficients of time and cross-correlation of the observation with the daily regime, were determined. The models were generated in two approaches: for all satellites and for individual blocks. To estimate the consistency and stability of the daily modeling solutions, the repeatability of the model parameters was tested. Table 3 contains the results of the repeatability of the determined measurement noise (for C/N${}_{0}=45$ dB-Hz) and the correlation coefficients for seven daily solutions calculated as the standard deviation from the determined parameters. The results, which are the maximum standard deviation values of a given system without distinguishing between satellite blocks, prove that the consistency of daily solutions is very high and, in most cases, does not exceed ±0.01 for time and cross-correlation coefficients as well as ±2 mm and ±0.01 mm for code and carrier-phase noise, respectively. Only in the case of code noise and carrier-phase correlation from GLONASS and BeiDou systems the repeatability is slightly lower. Therefore, the weekly solution calculated as the mean of the daily solutions was adopted as the final empirical model of variance and correlation of observations.

**Figure 22:**
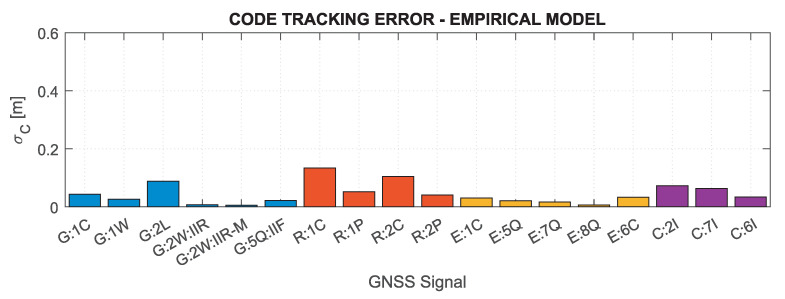
Empirical code tracking errors for GNSS signals for a receiver Septentrio PolaRx5.

**Figure 23:**
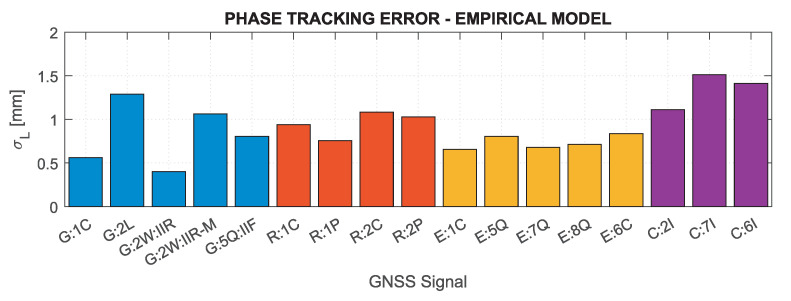
Empirical carrier-phase tracking errors for GNSS signals for a receiver Septentrio PolaRx5.

The weekly empirical measurement noise models for the minimum received signal power for individual code and carrier-phase GNSS signals are presented in Figure 22 and Figure 23. The values shown directly correspond to the nominal tracking errors (see Figure 2 and Figure 3) calculated for the same carrier-to-noise density ratio values. For easier comparison of empirical and nominal values, the corresponding figures scales have been retained, but for empirical values, there is no data for signals that were not tracked (GPS 2L and 5Q for block III and L1W; GLONASS 3Q). As can be easily seen, the empirical model largely coincides with the nominal values, especially for carrier-phase measurements, for which both the absolute values of errors and their mutual relations are highly consistent. Clear differences can be noticed only for the GPS L2W Block IIR signal, for which the empirical accuracy is three times higher than the nominal one. For code measurement errors, the observed differences are greater, particularly for the GPS 2L and GLONASS 1C and 2C signals, the empirical model gives much lower error values, which confirms the actual transmission bandwidth for these signals is greater than the nominal one. In general, the empirical code tracking errors are below the nominal values, but their mutual relation is close to the results obtained from theoretical analyses. The smallest errors, both for code and carrier-phase measurements, are characteristic for the Galileo signals; the highest are achieved for GLONASS and BeiDou.

### 5.2. Positioning Solution

The final weekly empirical stochastic model was evaluated based on solution results of the positioning model presented in Section 4. Positioning was performed for short baseline WUT1-WUT2 using 24-h data on a DOY 180, year 2018 which did not coincide with the data used to estimate the stochastic model. Raw 1 Hz code and carrier-phase measurement data was recorded using Septentrio PolaRx5 receivers connected to a Leica Choke-Ring AT504 antenna at WUT1 and a Septentrio PolaNt ChokeRing B3/E6 at WUT2. The ionosphere- and troposphere-fixed model, which assume that both delays are negligible for such a short baseline, was derived in a kinematic mode with an interval of 1 s, so 86,400 solutions were obtained in total. Although the short baseline observations are burdened with the multipath, which is not included in the positioning model, such a selection of test data allows estimating the effectiveness of the developed empirical stochastic model in real conditions that are never free from this phenomenon.

The modified Kalman Filter was used to determine the receiver position as well as float carrier-phase ambiguity. The parameters for tuning KF were set as follows: the variance of process noise was set to 1 m${}^{2}$/s for position components, nine cycle${}^{2}$/s for carrier-phase ambiguity as an initial value and $10^{-6}$ cycle${}^{2}$/s for subsequent epochs. The tuning parameters were adopted based on preliminary tests, assuming: kinematic solution for a receiver with the very low dynamics, large initial value of ambiguity process noise and a very low value for subsequent epochs [93,94]. The AR was performed applying the LAMBDA method [95]; the correctness of AR was verified in comparison with the true values computed based on the known coordinates of the WUT1 and WUT2 stations (called empirical success rate probability) as well as using a validation test based on theoretical probability of a success rate (its lower bound approximation) with a threshold not exceeding 0.99 [96]. The positioning model solution was performed in continuous mode, and the KF was reset to initial tuning parameters for ambiguity when cycle slips occurred or for all parameters when a number of estimated carrier-phase ambiguities dropped below six.

The impact of the developed empirical stochastic model on the positioning model solution was verified by comparing the results of the AR and the position estimation for several data processing scenarios: (a) the empirical model (denoted as “C/N${}_{0}$” model in Table 4, Table 5, Table 6, Table 7 and Table 8) vs. standard satellite elevation-dependent variance model (denoted as “Elev.”) for which the parameters $\sigma_{L}=0.003$ m +0.002 m/sine for carrier phase measurement and $\sigma_{C}=100\cdot \sigma_{L}$ for code measurement were adopted uniformly for all GNSS systems and signals; (b) the impact of using only variance parameters in both tested stochastic models vs. including also the determined time- and cross-correlation parameters; (c) the empirical models distinguishing between satellites blocks (denoted as “C/N${}_{0}$(b)”) vs. the unified model for all observations of a given signal; (d) using different combinations of GNSS measurements—single system GPS and GLONASS, dual system solution GPS + Galileo, GPS + BeiDou as well as Multi-GNSS approach using all systems. In all tested solutions, the observation cut-off mask was set to $5^{\circ }$ in case of using elevation-dependent stochastic model, and the 5th percentile of C/N${}_{0}$ in case of the empirical model, which corresponded to a very similar number of observations in both cases.

Table 4 presents the model solution results for tested scenarios for GPS observations. The AR outcomes are presented as the empirical success rate probability (expressed in percent) with the number of epochs (in parentheses) for which an incorrect AR was obtained. The AR validation test effectiveness was presented in a form of the epochs number for which the type I (false rejection, FR) and type II (false acceptance, FA) errors occurred. Positioning accuracy for fixed solutions has been presented in the form of Root Mean Square (RMS) errors for the horizontal (ne) and vertical (u) components, the percentage of epochs for which the 3D error in the relative reference position exceeds 5 mm and the maximum position error. In each of the tested scenarios, the empirical model results were better compared to the elevation model. In particular, the empirical model allowed for the correct AR for all epochs, which was impossible to achieve in the elevation model. Additionally, the positioning accuracy for the empirical model was apparently better. Incorporating the cross and temporal correlations into the model sequentially did not improve the positioning accuracy significantly, but it increased the effectiveness of the validation test for the elevation model. The best positioning performance was obtained for the individual satellite block models, but the difference is negligible compared to the uniform model. As an additional scenario, due to the small number of 5Q observations available, positioning was performed excluding these measurements. For such data, the best positioning results were obtained for the C/N${}_{0}$(b) model with errors ±3.0 mm and ±3.7 mm for horizontal and vertical components, errors below 5 mm at the level of 68% and a maximum error of 26.0 mm.

The results obtained for GLONASS (Table 5) essentially confirm the previous conclusions, although compared to GPS, the quality of the solution is lower. In each scenario, the use of the empirical model gives greater efficiency of AR and lower position errors, but only the use of a fully populated VC matrix in C/N${}_{0}$ model provides 100% effectiveness. A relatively large number of incorrect AR refers to the epochs in which observations from descending satellites were included in the model, which resulted in filter tuning taking several epochs. What is important, for the empirical model, the validation test incorrectly accepted all fixed ambiguities, which may indicate an overestimation of the measurement accuracy due to the presence of multipath, resulting in a decrease in the test significance level.

Due to the incomplete satellite constellation for Galileo and BeiDou, a dual-system solution GPS + Galileo (see Table 6) and GPS + BeiDou (see Table 7) was performed. In both cases, the 5Q signal was excluded from the GPS observation; also, the 5Q observations were excluded from the Galileo system as they are directly dependent on already used 8Q measurements. For BeiDou system, two types of empirical models were applied: the model distinguishing between satellite blocks (C/N${}_{0}$(b)) and the model distinguishing only geostationary satellites ( C/N${}_{0}$(G)). For both dual-system solutions, similar results were obtained, which indicate a clear improvement in the positioning accuracy and reliability with the use of empirical stochastic models. In each scenario, considering the additional correlation parameters in the elevation model increased the effectiveness of AR and reduced position errors. The empirical model allowed to achieve a higher accuracy of horizontal components (±1.9 mm) and a lower maximum error (16 mm) for GPS-Galileo solution. For the GPS-BeiDou, smaller height errors (±3.8 mm) and a much larger number of epochs with errors less than 5 mm (71%) were obtained. For tested approaches, the use of satellite-block-dependent models only slightly increased the solution quality.

The four system Multi-GNSS solution (Table 8) was performed for all signals, excluding GPS 5Q and Galileo 5Q measurements. For this test, the difference in the elevation model results compared to the empirical model was the greatest. The elevation model was characterized by the lowest efficiency of integer ambiguity estimation and validation compared to other experiments, and the efficiency increased along with including additional correlation parameters in the stochastic model. A dozen or so times higher number of incorrectly fixed ambiguities than in the single-system GPS solution proves that adopting the same stochastic parameters for all systems is erroneous and disrupts the solution reliability for Multi-GNSS positioning. The use of the empirical model ensures 100% effectiveness of AR and gives the lowest maximum errors (15.2 mm) among all tested scenarios.

**Figure 24:**
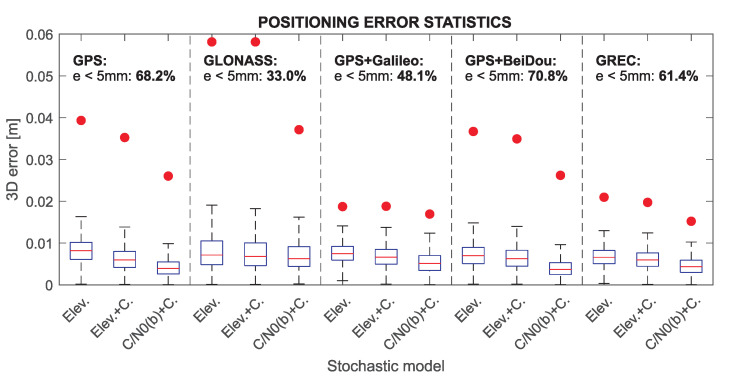
Positioning error statistics for different stochastic models and system combinations.

Figure 24 shows a summary of position errors for all tested scenarios for three stochastic approaches: the elevation-dependent model, the elevation model including the time- and cross-correlation (denoted as “Elev.+C.”) and individual for satellite blocks empirical model including correlation parameters (denoted as “C/N_0_(b)+C.”). The 3D position error statistics are shown in the form of a box plot, where a central mark of the box indicates the median, the top and bottom edges of the box mark the 75th and 25th percentiles of errors, and the whiskers are extended to the most extreme values of errors not considered outliers for a confidence level of approximately 99%. A red dot marks the maximum position error for each scenario. These statistics confirm that the positioning accuracy is increasing when considering the empirical stochastic parameters of measurement properties. The greatest accuracy characterizes the GPS and GPS + BeiDou solutions (with median 3.9 mm and 3.7 mm, respectively), while for the GPS + Galileo solution very low maximum errors were obtained (16.9 mm). The optimal compromise is the Multi-GNSS solution, where high accuracy (with median 4.3 mm) and the lowest maximum error (15.2 mm) are achieved.

## 6. Summary and Concluding Remarks

This contribution presents research on individual empirical stochastic models of Multi-GNSS measurements. Based on the theoretical analysis of the GNSS receiver tracking errors, which depend on the carrier-to-noise density ratio parameter, a variance model was proposed. The empirical parameters of the model were determined utilizing the measurement data from the zero-length and very short baselines. Comparing the nominal tracking errors for Septentrio receivers with those determined using the empirical model confirmed the high compliance of theoretical assumptions with the modeling results. Additionally, the zero-length baseline data time series analysis was used to determine the parameters of cross- and time-correlation of GNSS signals. Using the weekly solution for the empirical stochastic parameters, a fully populated variance-covariance matrix, individual for GNSS system, signal and satellite blocks, was created. The impact of empirical stochastic modeling on ambiguity resolution as well as on position accuracy was analyzed with respect to the standard elevation-dependent model for different positioning scenarios. Positioning models were derived using modified Kalman Filter in which time-correlation is taken into account. Based on the stochastic modeling and positioning results, we can conclude that:

- the C/N${}_{0}$-dependent stochastic model allows to consider the factors influencing the signal tracking errors and thus precisely modeling observation noise;
- the realistic empirical stochastic model increases the accuracy and reliability of positioning solution, in particular the effectiveness of ambiguity resolution, while including the correlation parameters increases this efficiency;
- the highest efficiency of applying the empirical model is noticeable for Multi-GNSS solutions, for which the adoption of uniform stochastic parameters for all systems leads to significantly lower reliability of the solution;
- the optimal solution in the context of AR, positioning accuracy and maximum errors was obtained for a four-system GNSS solution using a fully populated empirical model individual for satellite blocks.

It should be noted that the determined stochastic parameters, apart from the signal properties, are also closely related to the tested receivers and may differ depending on the equipment used. However, the presented methodology for creating empirical stochastic models and including them in the positioning model is comprehensive and can be used for all types of GNSS receivers and measurements. On the other hand, the bottleneck of the presented methodology may be the fact that the C/N${}_{0}$ parameter varies due to multipath, which in such a case may unreliably describe the precision of observations estimated based on triple-differences. In the case of strong multipath, the determined stochastic model should additionally consider the impact of this phenomenon. 

## Figures and Tables

**Table 1:** Signal power budget of GPS C/A for $e=5^{\circ }$ [7,79,80].

Component	Value
Transmitted signal power:	
Minimum signal power	14.3 dBW
Received signal power:	
Path loss	−159.0 dB/m${}^{2}$
Satellite antenna gain	12.4 dB
Atmospheric attenuation	2.0 dB
Receiver antenna gain	−27.4 dBm${}^{2}$
Received signal power	−161.8 dBW
Noise power:	
Noise power ($P_{N}$)	−141.0 dBW
Noise power density ($N_{0}$)	−204.0 dBW/Hz
Signal quality:	
Signal-to-noise ratio (SNR)	−20.8 dB
Carrier-to-noise density ratio (C/N${}_{0}$)	42.2 dB-Hz

**Table 2:** Expectation and dispersion values of DD and TD carrier-phase combination residuals for SB and ZB setup.

Obs.	Formula	E{·}	D{·}
SB:DD	$\nabla \Delta \tilde{L}\left(t_{1}\right)=\nabla \Delta L\left(t_{1}\right)-\nabla \Delta R\left(t_{1}\right)-\lambda \nabla \Delta N\left(t_{1}\right)$	$\nabla \Delta \delta M_{L}\left(t_{1}\right)$	$4\sigma_{L}^{2}$
SB:TD	$\nabla \Delta \tilde{L}\left(t_{12}\right)=\nabla \Delta \tilde{L}\left(t_{2}\right)-\nabla \Delta \tilde{L}\left(t_{1}\right)$	$\nabla \Delta \delta M_{L}\left(t_{2}\right)-\nabla \Delta \delta M_{L}\left(t_{1}\right)\approx 0$	$8\left(1-\rho_{t}\right)\sigma_{L}^{2}$
ZB:DD	$\nabla \Delta \tilde{L}\left(t_{1}\right)=\nabla \Delta L\left(t_{1}\right)-\lambda \nabla \Delta N\left(t_{1}\right)$	0	$4\left(1-\rho_{\Delta }\right)\sigma_{L}^{2}$
ZB:TD	$\nabla \Delta \tilde{L}\left(t_{12}\right)=\nabla \Delta \tilde{L}\left(t_{2}\right)-\nabla \Delta \tilde{L}\left(t_{1}\right)$	0	$8\left(1-\rho_{t}\right)\left(1-\rho_{\Delta }\right)\sigma_{L}^{2}$

**Table 3:** Repeatability of the stochastic model parameters for the weekly solution.

System	Noise (for C/N${}_{0}=45$ dB-Hz)		Time-Correlation		Cross-Correlation	
	$\sigma_{C}$ [mm]	$\sigma_{L}$ [mm]	$\sigma_{C}$	$\sigma_{L}$	$\sigma_{C}$	$\sigma_{L}$
GPS	±1.5	±0.01	±0.01	±0.01	±0.01	±0.01
GLONASS	±2.2	±0.01	±0.01	±0.04	±0.02	±0.03
Galileo	±1.2	±0.01	±0.01	±0.01	±0.01	±0.01
BeiDou	±5.2	±0.01	±0.01	±0.01	±0.01	±0.06

**Table 4:** Positioning model solution results for GPS.

GNSS	Stochastic Model			Ambiguity Resolution		Positioning Accuracy [mm]			
System	Model	Cross	Time	P succ. [%]	FA/FR	RMS (ne)	RMS (u)	Err. <5	Max (err.)
G	Elev.	–	–	99.9 (92)	31/2	3.3	8.3	15.1%	39.3
G	C/N${}_{0}$	–	–	100 (0)	0/0	2.7	5.0	54.6%	29.7
G	Elev.	√	–	99.9 (90)	29/2	3.3	8.5	13.8%	39.7
G	C/N${}_{0}$	√	–	100 (0)	0/0	2.7	4.9	54.9%	29.8
G	Elev.	√	√	99.9 (57)	10/14	3.3	8.3	16.3%	39.6
G	C/N${}_{0}$	√	√	100 (0)	0/0	3.0	4.6	55.9%	29.3
G	C/N${}_{0}$(b)	√	√	100 (0)	0/0	3.0	4.5	57.0%	28.9
G(-5Q)	Elev.	√	√	99.9 (50)	8/17	2.9	6.4	36.5%	35.3
G(-5Q)	C/N${}_{0}$	√	√	100 (0)	0/0	3.0	3.7	68.4%	26.4
G(-5Q)	C/N${}_{0}$(b)	√	√	100 (0)	0/0	3.0	3.7	68.2%	26.0

**Table 5:** Positioning model solution results for GLONASS.

GNSS	Stochastic Model			Ambiguity Resolution		Positioning Accuracy [mm]			
System	Model	Cross	Time	P succ. [%]	FA/FR	RMS (ne)	RMS (u)	Err. <5	Max (err.)
R	Elev.	–	–	99.7 (258)	127/4	4.7	8.9	27.1%	58.1
R	C/N${}_{0}$	–	–	99.8 (218)	218/0	4.4	7.3	31.5%	35.7
R	Elev.	√	–	99.6 (330)	214/4	4.7	8.8	27.2%	58.9
R	C/N${}_{0}$	√	–	99.9 (50)	50/0	4.5	7.4	30.8%	36.0
R	Elev.	√	√	99.6 (218)	129/5	4.5	8.6	29.5%	58.1
R	C/N${}_{0}$	√	√	100 (0)	0/0	4.4	7.2	32.6%	37.3
R	C/N${}_{0}$(b)	√	√	100 (0)	0/0	4.4	7.2	33.0%	37.1

**Table 6:** Positioning model solution results for GPS + Galileo.

GNSS	Stochastic Model			Ambiguity Resolution		Positioning Accuracy [mm]			
System	Model	Cross	Time	P succ. [%]	FA/FR	RMS (ne)	RMS (u)	Err. <5	Max (err.)
G(-5Q)E(-5Q)	Elev.	–	–	99.8 (128)	33/0	2.5	7.6	14.3%	18.7
G(-5Q)E(-5Q)	C/N${}_{0}$	–	–	100 (0)	0/0	2.0	6.3	33.8%	17.2
G(-5Q)E(-5Q)	Elev.	√	–	99.9 (124)	36/0	2.4	7.2	19.2%	18.7
G(-5Q)E(-5Q)	C/N${}_{0}$	√	–	99.9 (1)	1/0	1.9	5.5	48.1%	16.6
G(-5Q)E(-5Q)	Elev.	√	√	99.9 (100)	30/13	2.3	6.9	25.6%	18.8
G(-5Q)E(-5Q)	C/N${}_{0}$	√	√	100 (0)	0/0	1.9	5.7	46.8%	17.0
G(-5Q)E(-5Q)	C/N${}_{0}$(b)	√	√	100 (0)	0/0	1.9	5.6	48.1%	16.9

**Table 7:** Positioning model solution results for GPS + BeiDou.

GNSS	Stochastic Model			Ambiguity Resolution		Positioning Accuracy [mm]			
System	Model	Cross	Time	P succ. [%]	FA/FR	RMS (ne)	RMS (u)	Err. <5	Max (err.)
G(-5Q)C	Elev.	–	–	98.8 (1026)	963/0	2.9	7.3	23.9%	36.7
G(-5Q)C	C/N${}_{0}$(G)	–	–	100 (0)	0/0	2.4	4.2	66.6%	28.1
G(-5Q)C	Elev.	√	–	99.0 (892)	830/1	3.0	7.3	23.1%	36.8
G(-5Q)C	C/N${}_{0}$(G)	√	–	100 (0)	0/0	2.5	4.2	67.3%	27.9
G(-5Q)C	Elev.	√	√	99.6 (313)	265/9	2.8	6.7	32.1%	34.9
G(-5Q)C	C/N${}_{0}$(G)	√	√	100 (0)	0/0	2.6	3.8	70.6%	26.7
G(-5Q)C	C/N${}_{0}$(b)	√	√	100 (0)	0/0	2.5	3.8	70.8%	26.2

**Table 8:** Positioning model solution results for GPS + GLONASS + Galileo + BeiDou.

GNSS	Stochastic Model			Ambiguity Resolution		Positioning Accuracy [mm]			
System	Model	Cross	Time	P succ. [%]	FA/FR	RMS (ne)	RMS (u)	err. <5	Max (err.)
GREC	Elev.	–	–	98.2 (1567)	1352/0	2.5	6.8	24.2%	21.0
GREC	C/N${}_{0}$(G)	–	–	100 (0)	0/0	1.9	5.3	49.4%	15.9
GREC	Elev.	√	–	98.4 (1423)	1232/0	2.5	6.5	30.7%	19.7
GREC	C/N${}_{0}$(G)	√	–	100 (0)	0/0	1.9	4.7	60.8%	14.9
GREC	Elev.	√	√	99.7 (1145)	988/0	2.3	6.3	33.8%	19.7
GREC	C/N${}_{0}$(G)	√	√	100 (0)	0/0	1.9	4.8	60.6%	15.2
GREC	C/N${}_{0}$(b)	√	√	100 (0)	0/0	1.9	4.7	61.4%	15.2

## Data Availability

Not applicable.
